# Outcomes After Tonsillectomy in Children With Angelman Syndrome

**DOI:** 10.1177/19160216251364759

**Published:** 2025-08-08

**Authors:** Meera Chopra, Jennifer M. Siu, Erick Sell, Jane Summers, Jackie Chiang, Evan J. Propst, Evelina Pankiv, Nikolaus E. Wolter

**Affiliations:** 1 Department of Otolaryngology – Head and Neck Surgery, The Hospital for Sick Children, University of Toronto, Toronto, ON, Canada; 2 Division of Neurology, Children’s Hospital of Eastern Ontario, University of Ottawa, Ottawa, ON, Canada; 3 Department of Psychiatry, The Hospital for Sick Children, University of Toronto, Toronto, ON, Canada; 4 Division of Respiratory Medicine, Department of Paediatrics, The Hospital for Sick Children, University of Toronto, Toronto, ON, Canada; 5 Department of Anesthesia and Pain Medicine, The Hospital for Sick Children, University of Toronto, Toronto, ON, Canada

**Keywords:** Angelman syndrome, children, pain, postoperative complications, tonsillectomy

## Abstract

**Importance:**

Angelman syndrome is a rare genetic disorder characterized by developmental delay, sleep disturbances, and a happy demeanor. Tonsillectomies are common procedures for individuals with Angelman syndrome, and their postoperative recovery may be complicated by challenging pain assessments, respiratory complications, or feeding difficulties. Inappropriate laughing may mask perioperative pain and anxiety.

**Objective:**

The objective of this study is to review postoperative outcomes and complications of children with Angelman syndrome undergoing tonsillectomy.

**Methods (Design, Setting, Participants, Intervention, Measures):**

We conducted a retrospective review of patients with Angelman syndrome undergoing tonsillectomies from 2000 to 2024 in a quaternary pediatric hospital. Demographic, clinical, and surgical outcome variables were collected.

**Results:**

Twelve children with Angelman syndrome underwent tonsillectomy: 7 for sleep-disordered breathing, 4 for sialorrhea, and 1 for recurrent tonsillitis. Median (IQR) duration of stay was 4.0 (3.0-5.3) days. The most common reason for prolonged hospital stay was inadequate oral intake. Nine (75.0%) children experienced postoperative complications, most frequently pooling of secretions and oxygen desaturations. Three children (25.0%) experienced severe postoperative complications, including 1 opioid overdose, 1 respiratory distress, and 1 aspiration pneumonia. Two patients were readmitted to the hospital: 1 for irregular breathing and poor pain control, and 1 for epistaxis.

**Conclusion:**

The postoperative course following tonsillectomy in children with Angelman syndrome can be complicated by a prolonged recovery, inadequate pain control, opioid toxicity, respiratory complications, and poor oral intake. Caregiver input on pain behavior is critical to develop an effective postoperative management strategy.

**Relevance:**

Based on our results and a literature review, we have created recommendations for post-tonsillectomy care in children with Angelman syndrome.

## Graphical abstract

**Graphical Abstract:**
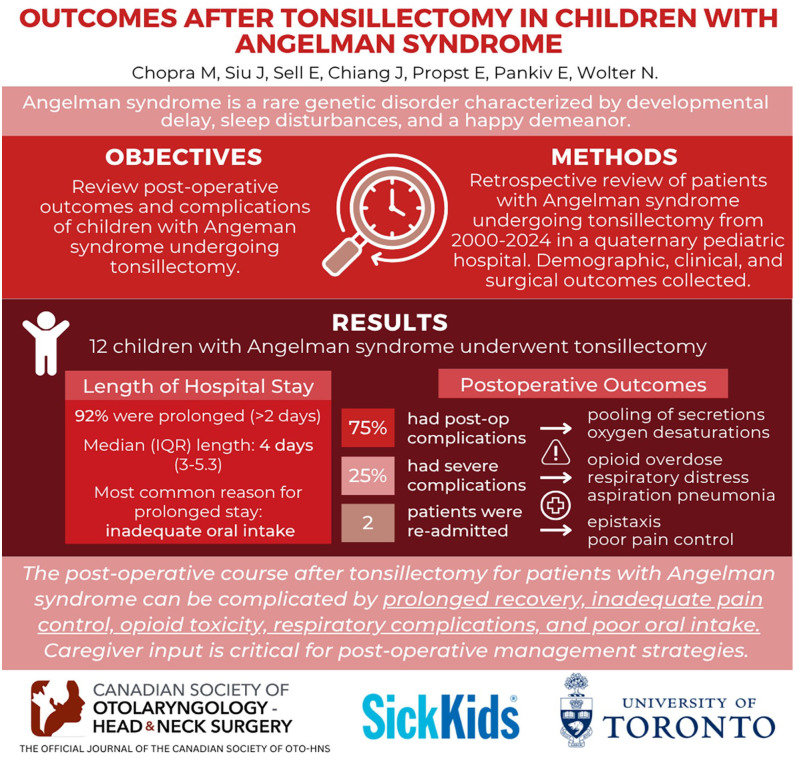

## Key Messages

- Tonsillectomies are common procedures for individuals with Angelman syndrome, a rare genetic disorder characterized by developmental delay and a happy demeanor.
- Their postoperative course may be complicated by a prolonged recovery, inadequate pain control, opioid toxicity, respiratory complications, and poor oral intake.
- Obtaining caregiver input on pain behavior is crucial to develop an effective postoperative pain management strategy.

## Introduction

Angelman syndrome (AS) is a rare genetic neurodevelopmental disorder with a prevalence of 1 in 12,000 to 1 in 20,000 people worldwide.^1^ It is caused by a microdeletion on the maternal copy of 15q11.2-q13, loss of function variants in the maternal *UBE3A* gene, uniparental paternal disomy, or imprinting center defects.^2^ These 4 molecular mechanisms all result in a lack of expression of *UBE3A* in the nervous system and dysfunction of the gamma-aminobutyric acid (GABA) receptor.^3,4^ Classically, children with AS present with a happy demeanor and frequent bouts of inappropriate laughter, movement or balance disorders, seizures, and a presumed high pain tolerance.^5^ Other common symptoms include severe developmental delay, sleep disturbances, absent or minimal speech, difficulty swallowing, and persistent sialorrhea.^6-8^ Given the clinical phenotype and comorbidities associated with AS, these children often require general anesthesia for routine medical procedures, such as dental extractions and electroencephalograms. Sleep disturbances are seen in up to 80% of children with AS.^9^ These can include problems with settling and sleep fragmentation, which may impair sleep quality, negatively impact behavior regulation, and worsen seizures.^10^ As such, many children with AS will be referred to an otolaryngologist’s office.

Tonsillectomy is one of the most common surgical procedures performed in children and can be associated with postoperative pain, decreased oral intake, dehydration, respiratory depression, and bleeding.^11^ Approximately 25% of individuals affected by AS receive a tonsillectomy or adenoidectomy in their lifetime, typically for sleep-disordered breathing or recurrent pharyngeal infections.^12,13^ Pain control may be a particular challenge in this population, given the consistently happy behavioral phenotype, presumed high pain tolerance, and lack of speech, which may complicate pain assessments.^5,14^ Patients with AS may also have underlying respiratory disorders or feeding difficulties, which may prevent adequate respiration, hydration, and feeding during their postoperative recovery.^15^

Given the wide range of comorbidities in patients with AS, the aim of this study was to review the perioperative course of children with AS who underwent a tonsillectomy in our quaternary care center with a focus on characterizing surgical outcomes and postoperative complications.

## Methods

A retrospective review was performed of children with AS who underwent tonsillectomy with or without adenoidectomy at The Hospital for Sick Children from January 1, 2000 to August 31, 2024. Cases were identified through a search for International Classification of Diseases 9th and 10th Revision codes for other deletions of part of a chromosome (Q93.5) and AS (Q93.51), and a free text search for “angelman” and “angel.” Patients were cross-referenced for having undergone tonsillectomy or adenoidectomy. Patients under the age of 18 with a confirmed diagnosis of AS who underwent a tonsillectomy for any clinical indication were then included in the review. This study was approved by the Hospital for Sick Children Research Ethics Board (REB #1000080352).

Demographic, clinical, and surgical characteristics were extracted from the electronic health records of each included patient. Clinical characteristics included the date of AS diagnosis, genetic records, previous medical diagnoses, presenting symptoms, and pre- and postoperative polysomnography, when available. Surgical characteristics included surgical indication, length of surgery, and surgical approach. Surgical outcome and complications were defined as length of hospital stay, pooling of secretions, oxygen desaturation, need for supplemental oxygen, poor oral intake, nausea or emesis, agitation, and fever. Summary of data using proportions, as well as medians and interquartile range (IQR), was performed using Microsoft Excel (Microsoft, 2025).

A literature review was conducted searching MEDLINE, Scopus, and Web of Science, using the following and related search terms: “Angelman,” “Angelman Syndrome,” “happy puppet,” “surgery,” “perioperative care,” “postoperative care,” and “anesthesia.” A gray literature search was also undertaken through an advanced Google search and rare disease-specific websites using the search terms “Angelman” and “surgery.”

We aimed to create practice recommendations for children with AS undergoing tonsillectomy using our experiences caring for patients within our institution and results from the literature review.

## Results

Twelve children with AS (25% female) underwent a tonsillectomy from 2000 to 2024. The median age at the time of surgery was 5.0 years (range: 1-9).

### Clinical Characteristics

All patients were diagnosed with AS postnatally. Four patients (33.3%) had a deletion on the maternally inherited chromosome 15q11.2-q13.1 region, 4 patients (33.3%) had a point mutation with the *UBE3A* region compatible with AS, 1 patient (8.3%) had a duplication within the *UBE3A* gene, 1 patient had uniparental disomy (8.3%), and 2 patients (16.7%) had an unknown genetic cause (Table 1).

**Table 1.** Demographic and Surgical Characteristics of Children Undergoing a Tonsillectomy.

Patient no.	Age at surgery (years) and sex	Genetic etiology of AS	Surgical indication	Surgical procedure	Time under anesthesia (min)	Operative time (min)	Surgical method	Length of hospital stay (days)
1	8F	Point mutation	Sialorrhea	Tonsillectomy + SMD relocation	165	135	Cold steel	5
2	8F	Point mutation	Sialorrhea	Tonsillectomy + SMD relocation	155	130	Cold steel	4
3	5M	Duplication	Sialorrhea	Tonsillectomy + SMD relocation	175	75	Cold steel	4
4	6F	Unknown	Sialorrhea	Tonsillectomy	47	17	Monopolar electrocautery	2
5	5M	Deletion	SDB	Tonsillectomy and adenoidectomy	91	68	Bipolar electrocautery	3
6	3M	Deletion	SDB	Tonsillectomy and adenoidectomy	48	37	Monopolar electrocautery	3
7	7M	Unknown	Recurrent tonsillitis	Tonsillectomy and adenoidectomy	50	20	Bipolar electrocautery	6
8	4M	Point mutation	SDB	Tonsillectomy and adenoidectomy	101	78	Monopolar electrocautery	4
9	4M	Point mutation	SDB	Tonsillectomy and adenoidectomy	112	92	Monopolar electrocautery	3
10	1M	Deletion	SDB	Tonsillectomy and adenoidectomy	153	94	Bipolar electrocautery	8
11	9M	Deletion	SDB	Tonsillectomy and adenoidectomy	88	48	Monopolar electrocautery	8
12	4M	Uniparental disomy	SDB	Tonsillectomy and adenoidectomy	94	55	Monopolar electrocautery	4

Abbreviations: AS, Angelman syndrome; SDB, sleep disorder breathing; SMD, submandibular duct.

The prevalence of common comorbidities in the AS population within our patients is described in Table 2.

**Table 2.** Prevalence of Certain Comorbidities in Patients With Angelman Syndrome.

Comorbidity	Prevalence, n (%)
Neurologic	
Developmental delay	12 (100)
Nonverbal	12 (100)
Seizures/Epilepsy	11 (91.7)
Sleep disturbances	10 (83.3)
Sleep-disordered breathing	7 (58.3)
Obstructive sleep apnea	2 (16.7)
Central sleep apnea	1 (8.3)
Ophthalmologic and otolaryngologic	
Sialorrhea	9 (75.0)
Strabismus	3 (25.0)
Respiratory	
Asthma	5 (41.7)
Aspiration pneumonia	4 (33.3)
Gastrointestinal	
Feeding difficulties	4 (33.3)
Jaundice	4 (33.3)
Constipation	2 (16.7)
Skeletal	
Ataxia or gait disturbances	10 (83.3)
Scoliosis	3 (25.0)

### Surgical Characteristics

Of the 12 surgeries, 7 (58.3%) were performed for sleep-disordered breathing, 4 (33.3%) for salivary duct rerouting to treat sialorrhea, and 1 (8.3%) for recurrent tonsillitis. Two children (#6 and 11) with sleep-disordered breathing had a formal diagnosis of obstructive sleep apnea (OSA) on polysomnography, with obstructive apnea-hypopnea indices (oAHI) of 5.0/hour and 4.0/hour, respectively. Eight patients (66.7%) underwent a tonsillectomy and adenoidectomy, 3 (25.0%) underwent tonsillectomy with submandibular duct relocation, and 1 (8.3%) underwent tonsillectomy alone (Table 1).

For the children who underwent a tonsillectomy and adenoidectomy, the median (IQR) time under anesthesia was 92.5 (78.5-103.8) minutes, and the median operative time was 61.5 (45.3-81.5) minutes. For the children who underwent tonsillectomy with submandibular duct relocation, the median (IQR) time under anesthesia was 165.0 (160.0-170.0) minutes, and the median operative time was 130.0 (102.5-132.5) minutes. The intraoperative course for most patients was uncomplicated, except for 1 patient (#10) who had a significant obstructive event during induction with an oxygen desaturation to 20%. This was attributed to secretions and laryngospasm. He recovered with mask ventilation, and the surgery proceeded without issue.

Three children (25.0%) received tonsillectomy with cold steel. Six children (50.0%) received monopolar electrocautery, and 3 children (25.0%) received bipolar electrocautery.

### Postoperative Course

All patients were transferred to a post-anesthesia care unit after surgery. During the immediate recovery period, 3 patients (#8, 9, and 10) experienced desaturations, one that resolved after a jaw thrust and coughing, one after mask ventilation and an oral airway placement, and the last after receiving supplemental blowby oxygen, respectively.

Inpatient postoperative complications are detailed in Table 3. There were 8 (66.7%) patients who had oxygen desaturation, of which 7 required supplemental oxygen therapy and one resolved spontaneously. Of the supplemental oxygen provision, blowby oxygen was the best tolerated, which 4 patients (33.3%) received. Three patients (25.0%) experienced a postoperative fever, 1 (8.3%) due to a respiratory tract infection, 1 (8.3%) due to suspected aspiration pneumonia, and 1 (8.3%) from an unclear cause. Eight patients (66.7%) experienced a pooling of oral secretions, 5 of which (41.7%) required frequent suctioning. These secretions appeared to be problematic for 2 patients—1 patient (#1) who had an oxygen saturation of approximately 83% to 86% on nasal prongs, which increased to 96% to 98% immediately after deep suctioning, and 1 patient (#10) who appeared to choke on these secretions.

**Table 3.** Postoperative Complications Experienced by Patients.

Symptom	Prevalence, n (%)
Pooling of secretions in the mouth or nose	8 (66.7)
Secretions requiring suctioning	5 (41.7)
Oxygen desaturations at any time <90%	8 (66.7)
Receipt of supplemental oxygen therapy	8 (66.7)
Blowby oxygen	4 (33.3)
Face mask	2 (16.7)
Nasal prongs	1 (8.3)
Nasopharyngeal tube	1 (8.3)
Poor oral intake	7 (58.3)
Nausea or emesis	5 (41.7)
Agitation	4 (33.3)
Fever	3 (25.0)

Children were then transferred to a dedicated pediatric otolaryngology ward. Eleven patients (91.7%) had a prolonged hospital stay of longer than 2 days, with the median (IQR) length of stay being 4 (3.0-5.3) days. Seven patients (58.3%) had a prolonged hospital stay due to inadequate oral intake, 2 (16.7%) had respiratory infections, 1 (8.3%) had a fever of unknown etiology, and 1 (8.3%) had an opioid overdose.

Three patients (#7, 10, and 11) experienced severe postoperative complications while in the hospital. Patient #7 experienced an opioid overdose 11 hours after surgery despite receiving an age-appropriate dose of morphine, requiring placement of a nasopharyngeal airway and supplemental oxygen. The child was treated with naloxone reversal and recovered well. Patient #10 had an upper respiratory tract infection on admission and then presented with a fever and hypothermia on postoperative days 3 and 6, respectively. He also experienced stridor and increased work of breathing, which improved with nebulized epinephrine. Patient #11 had a suspected aspiration pneumonia on postoperative day 1 and was treated with a 7-day course of antibiotics.

All patients were ultimately discharged home. Two patients (#5 and 12) were readmitted to the hospital following discharge. Patient #5 was readmitted 4 days after discharge due to irregular breathing, inadequate pain control, and poor oral intake. He was provided with more analgesia and discharged the following day. Patient #12 was readmitted 1 day after discharge due to epistaxis. He was monitored overnight and discharged the following day after the bleeding did not recur.

### Surgical Outcomes

Follow-up data were available for 9 (75.0%) patients. Patients were followed for a median (IQR) of 11.0 (6.0-17.0) months after their surgery. Three patients who received surgery to manage sialorrhea qualitatively noted significant improvements in their drooling, with 2 reporting that it was completely gone. Four patients with sleep-disordered breathing underwent postoperative polysomnography a median of 17.0 (range: 3.3-47.5) months after their tonsillectomy, which revealed a normal oAHI of less than 1.0/hour in all patients. One patient received both a pre- and postoperative polysomnogram, which showed a decrease in oAHI from 5.0/hour to 0.2/hour. One caregiver reported that a patient who did not undergo postoperative polysomnography had no apparent improvement in sleep 6 months after surgery, though overnight oximetry showed no significant desaturations.

### Literature Review

The literature search revealed 2 case reports and 2 case series, describing 7 patients with AS undergoing tonsillectomy and 1 patient undergoing submandibular duct reconstruction.^16-19^ Reports identified in our literature review noted that individualized anesthetic plans and postoperative observation are important for patients with AS, given complex comorbidities and unpredictable responses to anesthetics.^16-19^ Campero identified the post-anesthetic care unit as a particularly challenging area for children with AS and used an anticipatory strategy of quickly bringing caregivers to the bedside to identify pain-related behaviors.^17^ The majority of the cases had uncomplicated postoperative recovery periods. However, in the case series by Landsman et al, a 2-year-old undergoing adenotonsillectomy for OSA experienced stridor in the post-anesthesia care unit, requiring supplemental oxygen and epinephrine.^19^ She was later admitted to the pediatric intensive care unit and required intubation and ventilation on postoperative day 1. She was discharged from the hospital 20 days after surgery.

The gray literature search revealed caregiver reports of 2 patients with AS, one who received an adenotonsillectomy and one with a lingual tonsillectomy, both of whom were indicated for episodes of choking and swallowing difficulties.^20,21^ Postoperatively, the patient undergoing an adenotonsillectomy received supplemental oxygen, and the patient with the lingual tonsillectomy had poor oral intake. No severe postoperative complications were shared. Both patients were discharged from the hospital on postoperative day 1. The caregiver for the patient with feeding difficulties noted the importance of a feeding therapist and consuming soft foods to increase oral intake postoperatively.^21^

## Discussion

This case series summarizes the perioperative course of 12 children with AS undergoing a tonsillectomy with or without adenoidectomy at a quaternary pediatric hospital. The majority of postoperative recoveries were uncomplicated, though the children in our study had a longer hospital stay than neurotypically developing children.^22^

### Surgical Characteristics

Time in the operating room was prolonged for children with AS. This reflects, in part, the time required to support children during induction in general, but also the additional time to support those with increased behavioral needs. Surgical time was also lengthened by challenges with access due to positioning and craniofacial changes. At the end of surgery, extra time is generally spent ensuring hemostasis at our hospital, given our experience with the challenges of postoperative examination and management of ensuring postoperative bleeding. Considering all these factors, planning for additional surgical time may be considered for children at our institution.

In terms of surgical technique, recent literature has focused on the role of intracapsular versus total tonsillectomy to improve postoperative outcomes and reduce complications.^23^ Though there is no research specifically focused on the role of surgical technique in tonsillectomy in children with AS, several retrospective analyses have investigated outcomes and complications in similar conditions, such as Trisomy 21 and autism spectrum disorder.^24,25^ Mukerji et al found that children with developmental delay undergoing intracapsular tonsillectomy had a shorter length of stay and were less likely to receive postoperative narcotics, though parent-reported symptom burden was similar between the 2 techniques.^25^ Further research is needed to determine whether outcomes are similar within the AS population.

### Pain Management

Tonsillectomy is a painful surgery for all children, with pain being the most intense in the first 3 days after surgery before gradually decreasing.^22^ Assessing pain in children with AS may be complex, since their characteristic happy demeanor, severe developmental delay, and lack of speech may confound pain assessments. Laughter may increase markedly with anxiety and stress, and disruptive behaviors may have a communicative intent triggered by pain, fatigue, or anxiety.^26-29^ Warner et al noted that 3 patients with AS undergoing tonsillectomy did not receive analgesic medications during post-anesthesia recovery, nor were they reported to have pain.^16^ Given the intense, but predictable, course of pain associated with this surgery, the current suggested management of postoperative pain for all children involves scheduled provision of pain medications rather than on an as-needed basis, which depends on pain interpretation.^30^ Scheduled dosing may facilitate the delivery of adequate analgesia for patients with AS, since medications are given without relying on pain assessments that may be inaccurate.

Pain in patients with AS may present differently from that of typically developing children. Shaw et al described a patient with AS who presented with irritability, drowsiness, respiratory distress, and hand jerking after taking Tylenol #3, and thus, an opioid overdose was suspected.^31^ These symptoms were later attributed to a lack of adequate analgesia rather than an overdose. Other presentations for inadequate pain control may be poor oral intake, with patient #5 in our series being readmitted to the hospital for this reason, which caregivers attributed to difficulty with pain control. Several of our patients presented with agitation postoperatively, which may have also been related to pain. Having a caregiver at the bedside assisted in determining if their child was in pain and facilitated the provision of adequate analgesia.

Given the difficulties in ensuring proper pain management, determining a robust pain management strategy preoperatively with caregiver input, may ensure appropriate delivery of analgesia to children with AS. Customary pediatric pain rating scales, such as the Face, Legs, Activity, Cry, and Consolability scale, may not reflect AS patients’ true level of pain, given that they observe behaviors such as grimacing, crying, and consolability.^32^ Relying on typical physiologic indicators of pain, including tachycardia and hypertension, may be challenging given the possible high vagal tone that AS patients exhibit.^33^ Identifying how each child presents when they are in pain, according to caregiver reports, can ensure that healthcare providers are best prepared to intervene and provide analgesia. Separation anxiety is not uncommon in children with AS, and this could add to challenges before and after management.^34^ A preoperative anesthetic evaluation can be of benefit as well to create a patient-centered plan for induction and wake-up. Although it is felt that typical anesthetic techniques are safe, specific anesthetic considerations should be made for GABA receptor involvement in AS and atypical responses to benzodiazepines.^26,33^

### Opioid Usage

Opioid sparing strategies are desirable in children with OSA, since they are thought to have a higher sensitivity to their effects.^35^ With the goal of reducing postoperative opioid use, current guidelines recommend the use of both acetaminophen and nonsteroidal anti-inflammatory drugs for pain relief.^36^ However, a robust pain management strategy is crucial since postoperative pain control allows for functional recovery after surgery.^22,37^ Given that pain control is a challenge in children with AS, all patients in our study received postoperative opioids to manage their pain. Opioids are commonly used for analgesia during surgical procedures for children with AS without reported complications, which suggests their safety in this patient population.^18,38^

In our series, patient #7 experienced significant respiratory suppression despite receiving an appropriate dose of morphine for his age. He was noted to have a baseline low oxygen saturation and central sleep apnea (CSA). Since opioids have been associated with respiratory depression and central apnea, both the medication and these comorbidities may have contributed to the overdose.^39^

It is not uncommon for children with AS to have conditions affecting their respiratory drive. One study found that 3 of their 10 participants with AS were affected by OSA or CSA.^40^ Particularly in children with such comorbidities, it is important to continuously monitor for respiratory effort and adequate oxygenation, even when they are provided with an appropriate opioid dose.

### Respiratory Complications

Respiratory complications are not uncommon after tonsillectomy due to existing comorbidities, such as OSA, changes in muscular tone, and craniofacial changes combined with postoperative airway edema.^41,42^ In our study, 8 patients (66.7%) experienced a decrease in baseline oxygen saturation to less than 90% during their hospital stay, 7 of which (58.3%) occurred within the first 24 hours. However, in the general pediatric population, Kieran et al found that only 7.2% of children undergoing tonsillectomy had an oxygen desaturation below 90% in the first 24 hours after surgery. Their analysis revealed that a syndromic diagnosis, clinical diagnosis of OSA, and neurological diagnosis were all independent risk factors for desaturations.^41^ Lee et al found that in children with obesity, a lower O_2_ saturation nadir and time spent with oxygen saturation <90% on polysomnography were predictive of postoperative respiratory complications.^43^ In both of these studies, postoperative admission for monitoring of respiratory drive was recommended in children with these risk factors. Finally, an analysis of children with cerebral palsy, who have similar comorbidities as children with AS, found that respiratory complications after adenotonsillectomy were more common in individuals with increased oral dysfunction or decreased gross motor ability.^44,45^

Though no studies have specifically analyzed predictive factors for respiratory complications in children with AS, complications such as neurological disease, obesity, dysphagia, and movement disorders are common in this patient population, which may explain the high rate of desaturations within our cohort.^8^ However, further analysis to determine whether desaturations were the result of underlying AS or associated comorbidities is required. Regardless, we would recommend continuous oxygen saturation monitoring in patients with AS following tonsillectomy within the first 24 hours.

Overall, sleep disturbances are common in children with AS, and understanding how OSA contributes to this can be challenging.^9,26^ Overnight polysomnography is indicated for children suspected of having sleep-related breathing problems. However, since children with AS often have anxiety in unfamiliar settings, polysomnography can be a challenge. Future studies with video polysomnography may have a role in characterizing sleep disturbances in this patient population.

### Oral Intake

Poor oral intake is a common finding in the general pediatric population post-tonsillectomy. Previous findings suggest that only 55% of children undergoing a tonsillectomy will return to normal eating within 1 week after the procedure.^46^

Feeding difficulties are common within the AS population, typically related to challenges with oral motor coordination.^6^ Before tonsillectomy, 4 of our patients were noted to have feeding difficulty, whether related to poor oral motor function or low baseline oral intake. Postoperatively, 7 patients had difficulty with feeding, which may have been related to pre-existing comorbidities that were exacerbated by post-tonsillectomy pain. Oral motor coordination challenges and frequent sialorrhea may predispose children with AS to aspiration pneumonia.^16,26^ Patient #11 in our series had a suspected aspiration pneumonia during his course in the hospital, as well as silent aspiration of thin liquids on a follow-up videofluoroscopic swallow study. A review of oral intake and adjunctive feeding strategies needed prior to surgery is important to ensure safe feeding postoperatively. A low threshold to involve speech language pathology or occupational therapy for feeding concerns after tonsillectomy is advisable. In addition, for children with baseline swallowing difficulties, a modified diet with thick-consistency foods may be necessary to prevent aspiration.

Based on our experiences with children with AS undergoing tonsillectomy in our institution and a literature review, we have created a set of practice recommendations for their care in the hospital, detailed in Table 4.

**Table 4.** Summary of Postoperative Care Recommendations for Children With Angelman Syndrome.

Feature	Recommendation
Preoperative counseling	Prepare family for possible prolonged hospital stay. Offer a preanesthetic consultation to optimize medical health (eg, gastroesophageal reflux disease (GERD), seizures, and/or benzodiazepine intolerance).^19^
Pain management	Be aware that pain assessments may be confounded by behavioral disposition and use caregiver knowledge to understand known patient cues.^26^ Consider preoperative anesthetic evaluation to establish patient-centered care plan for induction and recovery. Determine a robust pain management strategy with caregivers preoperatively. Work with caregivers to identify unique pain presentations in each child.^14,17^ Caution around standard pain scales (eg, FLACC), which may emphasize typical pain behavior and consolability that may not apply.^32^ Consider tailoring pain indicators to behaviors noted by parents and physiological parameters.
Opioid usage	Provide standing acetaminophen and nonsteroidal anti-inflammatory drugs during the first 72 h with opioids for breakthrough pain. Monitor for respiratory effort and oxygenation in patients who receive opioids, regardless of dose.
Respiratory complications	Evaluate patients for sialorrhea to anticipate increased challenges with secretions postoperatively. Monitor breathing in the first 24 h after surgery.^41^ Consider preoperative polysomnography to anticipate respiratory challenges, recognizing that unfamiliar environments can be challenging, which may limit recordings. Given the dysfunction within the GABA receptor, atypical responses to benzodiazepines and other anesthetic agents modulated through this receptor are possible.^16,26^
Oral intake	Evaluate feeding strategies preoperatively. For children at risk of aspiration, offer thickened liquids and soft foods that do not require chewing postoperatively.

Abbreviation: FLACC, Face, Legs, Activity, Cry, and Consolability; GABA, gamma-aminobutyric acid.

### Limitations

The major limitation of this study is its small sample size, owing to the rarity of AS. To capture more patients, a long timeframe was used, and thus, our study included submandibular duct relocation, which is no longer a routine practice in our center. Although this procedure is no longer performed, tonsillectomy was performed in a similar way, and there were no clinical differences in the postoperative courses between these patients and those who underwent tonsillectomy. As well, follow-up data were not available for several patients, preventing a complete analysis of long-term outcomes. Finally, we have created a list of recommendations based on our experience in our institution and available literature (Table 4). Referenced articles are small and uncontrolled, but represent the best literature available. We believe our recommendations will provide clinicians with useful and evidence-based points for discussion, though these must be applied on an individual patient basis.

## Conclusion

The postoperative course of children with AS will likely include a prolonged recovery, and caregivers should be advised of this preoperatively. Certain complications to carefully monitor for in this patient population include untreated pain, opioid toxicity, respiratory complications, and difficulty with oral intake. Further studies are needed to conclusively establish the risks of this procedure and compare the risk of postoperative complications across patients with AS and neurotypically developing children.
